# Trichopolydesmidae from Cameroon, 2: A species-level reclassification of Afrotropical trichopolydesmids (Diplopoda, Polydesmida), with two new species and two new records from Cameroon, and two new species from the Nimba Mountains, Guinea

**DOI:** 10.3897/zookeys.891.46986

**Published:** 2019-11-21

**Authors:** Sergei I. Golovatch, Armand Richard Nzoko Fiemapong, Didier VandenSpiegel

**Affiliations:** 1 Institute for Problems of Ecology and Evolution, Russian Academy of Sciences, Moscow, Russia; 2 Laboratoire de Zoologie, Université Yaoundé 1, BP812, Cameroon; 3 Laboratory of Zoology, Higher Teacher’s Training College, Université Yaoundé I, P.O.Box 47, Yaounde, Cameroon; 4 Musée Royal de l'Afrique centrale, Tervuren, Belgium

**Keywords:** classification, millipede, new combination, new records, review, SEM iconography, taxonomy

## Abstract

A revised classification of Afrotropical Trichopolydesmidae is presented. The fauna presently contains as many as 52 species in six recognized genera, with numerous new transfers/combinations involved: *Bactrodesmus* Cook, 1896 (3 species, including *B. grandis***sp. nov.** from the Nimba Mountains, Guinea), *Eburodesmus* Schubart, 1955 (2 species), *Hemisphaeroparia* Schubart, 1955 (26 species, including one old species, *Polydesmus parvulus* Porat, 1894, revised from type material and provisionally assigned to *Hemisphaeroparia*, as well as two new records and two new species from Cameroon: *H. longibrachiata***sp. nov.** and *H. avis***sp. nov.**), *Mecistoparia* Brolemann, 1926 (3 species), *Physetoparia* Brolemann, 1920 (12 species, including *P. complexa***sp. nov.** from the Nimba Mountains, Guinea), and *Sphaeroparia* Attems, 1909 (6 species). The hitherto enigmatic genus *Bactrodesmus* is redefined, but the monotypic *Trichozonus* Carl, 1905 still remains dubious.

## Introduction

All Afrotropical genera of the millipede family Trichopolydesmidae have recently been reviewed based on their type species and a presumed scenario of gonopodal evolution (Golovatch et al. 2018). As a result, in addition to two still enigmatic genera, *Bactrodesmus* Cook, 1896 and *Trichozonus* Carl, 1905, only five genera have been regarded as currently recognizable: *Sphaeroparia* Attems, 1909, *Physetoparia* Brolemann, 1920, *Eburodesmus* Schubart, 1955, *Mecistoparia* Brolemann, 1926 and *Hemisphaeroparia* Schubart, 1955. The last genus listed is the sole trichopolydesmid to occur in Cameroon and is especially diverse (26 species).

The present contribution provides a species-level reclassification of Afrotropical Trichopolydesmidae and gives a new diagnosis of *Bactrodesmus* based on the discovery of a new species from the Nimba Mountains, Guinea. Two additional records and two new species of *Hemisphaeroparia* are described from Cameroon, while the sole old and still enigmatic species reported from that country, *Polydesmus parvulus* Porat, 1894, is revised from female syntypes and is tentatively assigned to *Hemisphaeroparia* as well. A new species of *Physetoparia* is also described from the Nimba Mountains, Guinea.

## Material and methods

Most of the material treated here derives from the collection of the Musée Royal de l’Afrique Centrale (**MRAC**), Tervuren, Belgium, with only a few duplicates retained for the collections of the University of Yaounde 1 (**UY1**), Cameroon or donated to the Zoological Museum, State University of Moscow (**ZMUM**), Russia, as indicated below. The samples are stored in 70% ethanol. Specimens for scanning electron microscopy (SEM) were air-dried, mounted on aluminium stubs, coated with gold and studied using a JEOL JSM-6480LV scanning electron microscope. The colour pictures were taken using the focus stacking setup described by Brecko et al. (2014). Canon EOS Utility software was used to control the camera. Zerene Stacker was applied for stacking the individual pictures into one ‘stacked image’.

The abbreviations used to denote gonopodal structures are explained directly in the text and figure captions.

## Results

### A revised list of Afrotropical Trichopolydesmidae, arranged in alphabetic order

***Bactrodesmus* Cook, 1896**

1. *Bactrodesmus bicornis* (Demange & Mauriès, 1975), Mount Tonkoui, Côte d’Ivoire (Demange and Mauriès 1975), originally described as *Sphaeroparia bicornis* Demange & Mauriès, 1975. Because it shows strongly enlarged ♂ legs 2 and 3, it definitely belongs to *Bactrodesmus*, thus representing a comb. nov. ex *Sphaeroparia*.

2. *Bactrodesmus claviger* Cook, 1896, the type species by subsequent monotypy, Liberia (Cook 1896b).

3. *Bactrodesmus grandis* sp. nov., Nimba Mountains, Guinea (see below).

***Eburodesmus* Schubart, 1955**

1. *Eburodesmus cyrtus* Schubart, 1955, Mount Tonkoui, Côte d’Ivoire (Schubart 1955).

2. *Eburodesmus erectus* Schubart, 1955, the type species by original designation, Guinea and Côte d’Ivoire (Schubart 1955).

### *Hemisphaeroparia* Schubart, 1955

1. *Hemisphaeroparia avis* sp. nov., Cameroon (see below).

2. *Hemisphaeroparia bamboutos* Golovatch, Nzoko Fiemapong, Tamesse, Mauriès & VandenSpiegel, 2018, Cameroon (Golovatch et al. 2018).

3. *Hemisphaeroparia bangoulap* Golovatch, Nzoko Fiemapong, Tamesse, Mauriès & VandenSpiegel, 2018, Cameroon (Golovatch et al. 2018).

4. *Hemisphaeroparia boletiphora* (Mauriès, 1968), Gabon (Mauriès 1968). Originally described as Mecistoparia (Mabocus) boletiphora Mauriès, 1968, it definitely belongs to *Hemisphaeroparia* as it shows not only ♂ epicranial modifications and conspicuously enlarged spiracles next to coxa 1 or 2, but also clearly enlarged and globose gonocoxae, the telopodites being strongly sunken inside a deep gonocoel and leaving at least two exposed branches (Golovatch et al. 2018). This formally results in the following new transfer: *Hemisphaeroparia boletiphora* (Mauriès, 1968), comb. nov. ex *Mecistoparia*.

5. *Hemisphaeroparia bonakanda* Golovatch, Nzoko Fiemapong, Tamesse, Mauriès & VandenSpiegel, 2018, Cameroon (Golovatch et al. 2018).

6. *Hemisphaeroparia cumbula* Schubart, 1955, the type species by original designation, Nimba Mountains, Guinea and Mount Tonkoui, Côte d’Ivoire (Schubart 1955).

7. *Hemisphaeroparia digitifer* Golovatch, Nzoko Fiemapong, Tamesse, Mauriès & VandenSpiegel, 2018, Cameroon (Golovatch et al. 2018).

8. *Hemisphaeroparia falcata* Golovatch, Nzoko Fiemapong, Tamesse, Mauriès & VandenSpiegel, 2018, Cameroon (Golovatch et al. 2018, see also below).

9. *Hemisphaeroparia fusca* Golovatch, Nzoko Fiemapong, Tamesse, Mauriès & VandenSpiegel, 2018, Cameroon (Golovatch et al. 2018).

10. *Hemisphaeroparia galeata* (Mauriès, 1968), Gabon (Mauriès 1968). Originally described as Mecistoparia (Mabocus) galeata Mauriès, 1968, it definitely belongs to *Hemisphaeroparia* as it shows not only ♂ epicranial modifications and conspicuously enlarged spiracles next to coxa 1 or 2, but also clearly enlarged and globose gonocoxae, the telopodites being strongly sunken inside a deep gonocoel and leaving at least two exposed branches (Golovatch et al. 2018). This formally results in the following new transfer: *Hemisphaeroparia galeata* (Mauriès, 1968), comb. nov. ex *Mecistoparia*.

11. *Hemisphaeroparia guerouti* Demange, 1967, Côte d’Ivoire (Demange 1967). Mauriès and Heymer (1996) transferred this species to *Sphaeroparia*, but we return it to *Hemisphaeroparia* herewith.

12. *Hemisphaeroparia hallini* (Demange & Mauriès, 1975), Mount Tonkoui, Côte d’Ivoire Demange and Mauriès 1975). Originally described as *Sphaeroparia hallini* Demange & Mauriès, 1975, but it seems to fit better in *Hemisphaeroparia* because of enlarged and globose gonocoxae, coupled with each telopodite being strongly sunken inside a deep gonocoel and leaving one rather long branch partly exposed (Golovatch et al. 2018). This results in the following formal transfer: *Hemisphaeroparia hallini* (Demange & Mauriès, 1975), comb. nov. ex *Sphaeroparia*.

13. *Hemisphaeroparia hexatricha* (Mauriès & Heymer, 1996), Kivu, the Democratic Republic of the Congo (Mauriès and Heymer 1996). Originally described as Sphaeroparia (Physetoparia) hexatricha Mauriès & Heymer, 1996, it seems best to assign to *Hemisphaeroparia* because of enlarged and globose gonocoxae, coupled with each telopodite being strongly sunken inside a deep gonocoel and leaving one rather long branch clearly exposed (Golovatch et al. 2018). This results in the following formal transfer: *Hemisphaeroparia hexatricha* (Mauriès & Heymer, 1996), comb. nov. ex *Sphaeroparia*.

14. *Hemisphaeroparia integrata* (Porat, 1894), Cameroon (Porat 1894; Golovatch et al. 2018). This species was originally described as *Polydesmus integratus* Porat, 1894, but Golovatch et al. (2018), based on a revision of the ♂ holotype, redescribed and transferred it to *Hemisphaeroparia*.

15. *Hemisphaeroparia longibrachiata* sp. nov., Cameroon (see below).

16. *Hemisphaeroparia mouanko* Golovatch, Nzoko Fiemapong, Tamesse, Mauriès & VandenSpiegel, 2018, Cameroon (Golovatch et al. 2018).

17. *Hemisphaeroparia nyabitabae* (Mauriès & Heymer, 1996), Ruwenzori Mts, Uganda (Mauriès and Heymer 1996). Originally described as Sphaeroparia (Physetoparia) nyabitabae Mauriès & Heymer, 1996, it seems best to assign to *Hemisphaeroparia* because of enlarged and globose gonocoxae, coupled with each telopodite being strongly sunken inside a deep gonocoel and leaving one rather long branch clearly exposed (Golovatch et al. 2018). This results in the following formal transfer: *Hemisphaeroparia nyabitabae* (Mauriès & Heymer, 1996), comb. nov. ex *Sphaeroparia*.

18. *Hemisphaeroparia ongot* Golovatch, Nzoko Fiemapong, Tamesse, Mauriès & VandenSpiegel, 2018, Cameroon (Golovatch et al. 2018).

19. *Hemisphaeroparia parva* Golovatch, Nzoko Fiemapong, Tamesse, Mauriès & VandenSpiegel, 2018, Cameroon (Golovatch et al. 2018).

20. *Hemisphaeroparia parvocristata* (Mauriès, 1968), Gabon (Mauriès 1968). Originally described as Mecistoparia (Mabocus) parvocristata Mauriès, 1968, it definitely belongs to *Hemisphaeroparia* as it shows not only ♂ epicranial modifications and conspicuously enlarged spiracles next to coxa 1 or 2, but also clearly enlarged and globose gonocoxae, the telopodites being strongly sunken inside a deep gonocoel and leaving at least two exposed branches (Golovatch et al. 2018). This formally results in the following new transfer: *Hemisphaeroparia parvocristata* (Mauriès, 1968), comb. nov. ex *Mecistoparia*.

21. *Hemisphaeroparia parvula* (Porat, 1894), Cameroon (Porat 1894; Golovatch et al. 2018). This species was originally described as *Polydesmus parvulus* Porat, 1894, but Golovatch et al. (2018) tentatively transferred it to *Hemisphaeroparia*. Based on a revision of both ♀ syntypes, this combination is confirmed here (see below).

22. *Hemisphaeroparia pileata* (Mauriès, 1968), Gabon (Mauriès 1968). Originally described as Mecistoparia (Mabocus) pileata Mauriès, 1968, it definitely belongs to *Hemisphaeroparia* as it shows not only ♂ epicranial modifications and conspicuously enlarged spiracles next to coxa 1 or 2, but also clearly enlarged and globose gonocoxae, the telopodites being strongly sunken inside a deep gonocoel and leaving at least two exposed branches (Golovatch et al. 2018). This formally results in the following new transfer: *Hemisphaeroparia pileata* (Mauriès, 1968), comb. nov. ex *Mecistoparia*.

23. *Hemisphaeroparia pretzmanni* (Demange & Mauriès, 1975), Mount Tonkoui, Côte d’Ivoire (Demange and Mauriès 1975). Originally described as *Sphaeroparia pretzmanni* Demange & Mauriès, 1975, but it seems to fit best in the genus *Hemisphaeroparia* because of clearly showing enlarged and globose gonocoxae, coupled with each telopodite being strongly sunken inside a deep gonocoel and leaving one rather long branch partly exposed (Golovatch et al. 2018). This results in the following formal transfer: *Hemisphaeroparia pretzmanni* (Demange & Mauriès, 1975), comb. nov. ex *Sphaeroparia*.

24. *Hemisphaeroparia spiniger* Golovatch, Nzoko Fiemapong, Tamesse, Mauriès & VandenSpiegel, 2018, Cameroon (Golovatch et al. 2018, see also below).

25. *Hemisphaeroparia subfalcata* Golovatch, Nzoko Fiemapong, Tamesse, Mauriès & VandenSpiegel, 2018, Cameroon (Golovatch et al. 2018).

26. *Hemisphaeroparia zamakoe* Golovatch, Nzoko Fiemapong, Tamesse, Mauriès & VandenSpiegel, 2018, Cameroon (Golovatch et al. 2018).

***Mecistoparia* Brolemann, 1926**

1. *Mecistoparia cristata* Brolemann, 1926, Benin (Brolemann 1926).

2. *Mecistoparia lophotocrania* Brolemann, 1926, the type species by original designation, Benin (Brolemann 1926).

3. *Mecistoparia pusilla* (Verhoeff, 1941), the type species of *Dendrobrachypus* Verhoeff, 1941 by monotypy, Fernando Po (Verhoeff 1941). Since the synonymization of both genera by Golovatch et al. (2018), the new transfer can be formalized as follows: *Mecistoparia pusilla* (Verhoeff, 1941), comb. nov. ex *Dendrobrachypus*.

***Physetoparia* Brolemann, 1920**

1. *Physetoparia beshkovi* (Mauriès & Heymer, 1996), Ruwenzori Mts, Uganda (Mauriès and Heymer 1996). Originally described as Sphaeroparia (Sphaeroparia) beshkovi Mauriès & Heymer, 1996, it actually belongs to *Physetoparia* as redefined by Golovatch et al. (2018): both gonopodal coxae and gonocoel medium-sized; telopodite usually less strongly exposed and less complex (when strongly exposed, then with a protective coxal apicolateral process), with two strong branches; seminal groove short and simple, solenomere relatively long, subspiniform. This results in the following formal transfer: *Physetoparia beshkovi* (Mauriès & Heymer, 1996), comb. nov. ex *Sphaeroparia*.

2. *Physetoparia complexa* sp. nov., Nimba Mountains, Guinea (see below).

3. *Physetoparia difficilis* (Kraus, 1958), the Democratic Republic of the Congo (Kraus 1958). Since the synonymization of *Mabocus* Chamberlin, 1951 with *Physetoparia* by Golovatch et al. (2018), the species must be referred to as *Physetoparia difficilis* (Kraus, 1958), comb. nov. ex *Mabocus*.

4. *Physetoparia edentula* (Attems, 1953), Kivu, the Democratic Republic of the Congo (Attems 1953). Originally described as *Elgonicola edentula*, since the synonymization of *Elgonicola* with *Physetoparia* by Golovatch et al. (2018), it must be referred to as *Physetoparia edentula* (Attems, 1953), comb. nov. ex *Elgonicola*.

5. *Physetoparia granulifer* (Chamberlin, 1951), the type species of *Mabocus* Chamberlin, 1951 by original designation, Angola (Chamberlin 1951; Kraus 1958). Since the synonymization of *Mabocus* with *Physetoparia* by Golovatch et al. (2018), the species must be referred to as *Physetoparia granulifer* (Attems, 1953), comb. nov. ex *Mabocus*.

6. *Physetoparia imbecilla* (Brolemann, 1920), the type species by monotypy, Mount Kinangop, Kenya (Brolemann 1920). Originally described as Sphaeroparia (Physetoparia) imbecilla Brolemann, 1920, it is to be referred to as *Physetoparia imbecilla* (Brolemann, 1920), comb. nov.

7. *Physetoparia jeanneli* (Attems, 1939), Mount Elgon, Uganda (Attems 1939). This is the type species of *Elgonicola* Attems, 1939 by original designation, the genus synonymized by Golovatch et al. (2018), formally resulting in *Physetoparia jeanneli* (Attems, 1939), comb. nov. ex *Elgonicola*.

8. *Physetoparia microchaeta* (Attems, 1939), Mount Elgon, Uganda (Attems 1939). Originally described as a subspecies of *Elgonicola jeanneli*, but the striking difference in the length of tergal setae between the two subspecies, let alone their strict sympatry (Mount Elgon) correctly allowed Mauriès and Heymer (1996) to elevate the rank of *microchaeta* to full species, formally resulting in *Physetoparia microchaeta* (Attems, 1939), comb. nov. ex *Elgonicola*.

9. *Physetoparia petarberoni* (Mauriès & Heymer, 1996), Ruwenzori Mts, Uganda (Mauriès and Heymer 1996). Originally described as Sphaeroparia (Sphaeroparia) petarberoni Mauriès & Heymer, 1996, it actually belongs to *Physetoparia* as redefined by Golovatch et al. (2018): both gonopodal coxae and gonocoel medium-sized; telopodite usually less strongly exposed and less complex (when strongly exposed, then with a protective coxal apicolateral process), with one strong branch; seminal groove short and simple, solenomere relatively short and subspiniform. This results in the following formal transfer: *Physetoparia petarberoni* (Mauriès & Heymer, 1996), comb. nov. ex *Sphaeroparia*.

10. *Physetoparia sangae* (Chamberlin, 1951), Angola (Chamberlin 1951; Kraus 1958). Since the synonymization of *Mabocus* Chamberlin, 1951 with *Physetoparia* by Golovatch et al. (2018), the species must be referred to as *Physetoparia sangae* (Attems, 1953), comb. nov. ex *Mabocus*.

11. *Physetoparia villiersi* (Schubart, 1955), the type species *Heterosphaeroparia* Schubart, 1955 by original designation, Nimba Mountains, Guinea and Mount Tonkoui, Côte d’Ivoire (Schubart 1955; Demange and Mauriès 1975). This species was originally described in *Heterosphaeroparia* Schubart, 1955, then relegated to *Sphaeroparia* (Demange and Mauriès 1975; Mauriès and Heymer 1996), but since the synonymization of *Heterosphaeroparia* with *Physetoparia* by Golovatch et al. (2018), it must be transferred to *Physetoparia*, comb. nov. ex *Sphaeroparia*.

12. *Physetoparia violantennae* (Mauriès & Heymer, 1996), Ruwenzori Mts, Uganda (Mauriès and Heymer 1996). Originally described as Sphaeroparia (Sphaeroparia) violantennae Mauriès & Heymer, 1996, it actually belongs to *Physetoparia* as redefined by Golovatch et al. (2018): both gonopodal coxae and gonocoel medium-sized; telopodite strongly exposed, but less complex, with a large apicolateral lobe, one strong branch and a strong spiniform solenomere. This results in the following formal transfer: *Physetoparia violantennae* (Mauriès & Heymer, 1996), comb. nov. ex *Sphaeroparia*.

***Sphaeroparia* Attems, 1909**

1. *Sphaeroparia attenuata* Brolemann, 1920, Mount Kilimanjaro, Tanzania (Brolemann 1920). Originally described as a subspecies of *minuta* (see below), but the differences noted by Brolemann (1920) between the two subspecies, especially those in the proportions and shapes of the various outgrowths of the gonopodal telopodites, allowed Mauriès and Heymer (1996) to correctly regard *attenuata* as a distinct species.

2. *Sphaeroparia lanceolata* Brolemann, 1920, Mount Kenya, Kenya (Brolemann 1920).

3. *Sphaeroporia lignivora* Brolemann, 1920, the type species of *Megaloparia* Brolemann, 1920 by subsequent designation by Attems (1940), Mount Kenya, Kenya (Brolemann 1920). *Megaloparia* has been synonymized with *Sphaeroporia* by Mauriès and Heymer (1996).

4. *Sphaeroparia minuta* Attems, 1909, the type species by monotypy, Mount Meru, Tanzania (Attems 1909).

5. *Sphaeroparia pygmaea* Brolemann, 1920, Shimoni, Kenya (Brolemann 1920). Originally described as Sphaeroparia (Megaloparia) pygmaea, but *Megaloparia* has been synonymized with *Sphaeroporia* by Mauriès and Heymer (1996).

6. *Sphaeroparia uncinata* Brolemann, 1920, Mount Kenya, Kenya (Brolemann 1920).

The above list contains 52 species, including 26 in *Hemisphaeroparia*, 12 in *Physetoparia*, six in *Sphaeroparia*, three each in *Mecistoparia* and *Bactrodesmus*, and two in *Eburodesmus*. One more species remains in the dubious genus *Trichozonus* (see below). We describe here another four new species in three genera and clarify the identity of *Bactrodesmus*. Additional records of two species recently described from Cameroon are also presented.

### Species descriptions

#### Physetoparia complexa sp. nov.

Taxon classificationAnimaliaPolydesmidaFuhrmannodesmidae

CD6F8805-CC23-5696-8EFD-57222950177C

http://zoobank.org/C6BD407F-E50B-495E-A0E7-0402D0008563

Figs 1A , 2 , 3

##### Type material.

***Holotype*** ♂ (MRAC 22840), Guinea, Nimba Mountains, summit of Mount Nion, ca 1405 m a.s.l., forest litter, 28.V.2019, A. Henrard, D. VandenSpiegel, C. Allard et al. leg. (Nimba 2019-24). ***Paratypes***: 1 ♂ (MRAC 22841), 9 ♀ (MRAC 22852), 1 ♂ (SEM, MRAC 22842), same locality and date, together with holotype.

##### Diagnosis.

Differs from all other species of the genus by the unusually complex gonopodal structure, i.e. the presence of a particularly prominent, distolateral, gonocoxal lobe (lo) that protects a similarly clearly exposed telopodite, the latter being largely represented by a high apicomesal lobe/outgrowth (ab) that carries a highly peculiar, large, tube-shaped solenomere (tu). The gonocoel is shallow and conceals only the bases of the telopodites (Figs 2K, 3).

##### Name.

To emphasize the complex gonopodal structure; adjective in feminine gender.

##### Description.

**Figure 1.**
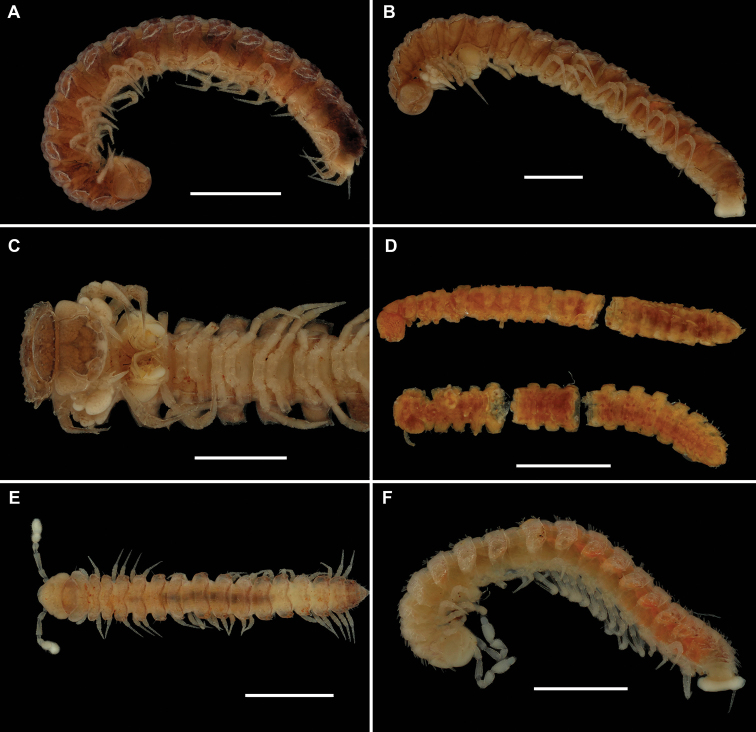
Habituses of **A***Physetoparia complexa* sp. nov., ♂ holotype, lateral view **B, C***Bactrodesmus grandis* sp. nov., ♂ paratype, lateral view of entire animal and its anterior body half, ventral view **D***Hemisphaeroparia parvula* (Porat, 1894), both ♀ syntypes, lateral, subventral or sublateral view **E***Hemisphaeroparia longibrachiata* sp. nov., ♂ holotype, dorsal view **F***Hemisphaeroparia avis* sp. nov., ♂ paratype, lateral view. Scale bars: 1.0 mm.

Length of holotype ca 5 mm (♂), width of midbody pro- and metazonae 0.5 and 0.7 mm (♂), respectively. Length of paratypes ca 5 mm (♂) or 6–7 mm (♀), width of midbody pro- and metazonae 0.5 and 0.7 mm (♂) or 0.6–0.7 and 0.8–1.0 mm (♀), respectively. Coloration in alcohol marbled light or darker reddish brown, venter and legs light brown to nearly pallid (Fig. 1A).

**Figure 2.**
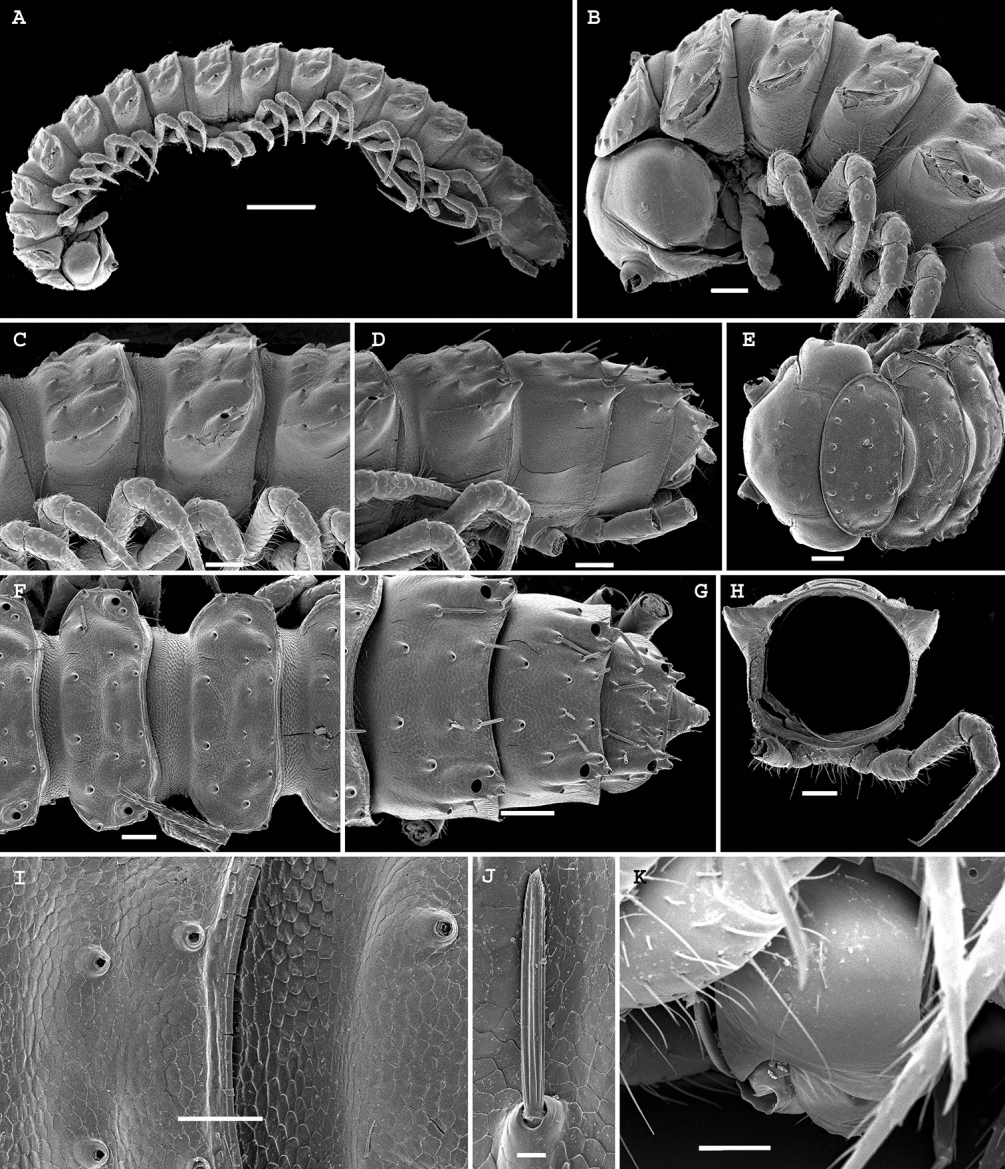
*Physetoparia complexa* sp. nov., SEM micrographs of ♂ paratype **A** habitus, lateral view **B, E** anterior part of body, lateral and dorsofrontal views, respectively **C, F** midbody segments, lateral and dorsal views, respectively **D, G** posterior part of body, lateral and dorsal views, respectively **H** cross-section of a midbody segment, caudal view **I** fine tergal structure, dorsal view **J** tergal seta, lateral view **K** gonopodal coxa in situ, lateral view. Scale bars: 0.5 mm (**A**), 0.1 mm (**B–H**), 0.05 mm (**I, K**), 0.01 mm (**J**).

Body with 20 segments in both sexes. Tegument very delicately micro-alveolate, mainly slightly shining. Head densely micropilose, devoid of epicranial modifications (Fig. 2A, B, E). Interantennal isthmus almost two times diameter of antennal socket. Antennae long and strongly clavate, reaching back past segment 3 when stretched dorsally. In length, antennomere 3 = 6 > 2 = 5 > 1 = 4 =7; antennomere 6 the largest, antennomeres 5 and 6 each with a distinct, round, distodorsal field of sensilla. In width, collum < head < segments 2–4 < 5–16; thereafter body gradually tapering towards telson. Collum ellipsoid, transversely oval, like all following metaterga with three transverse, regular rows of setae on low, but evident, setigerous bosses. Tergal setae medium-sized, each ca 1/4–1/5 as long as metatergum, bacilliform and longitudinally ribbed, gradually growing longer towards telson, set on minute knobs (Fig. 2A–J), always 3+3 in each row on postcollum metaterga; 2–3 additional setae normally present at lateral margin of paraterga. A faint, sinuate, transverse sulcus visible behind first row on most metaterga. Dorsum invariably regularly convex. Paraterga medium-sized, set at around upper 1/3 of metazonae (Fig. 2A–H), visible starting with collum, often slightly upturned caudally, faintly, but regularly rounded and bordered, lateral incisions almost absent. Caudal corner of paraterga mostly rounded, sharply truncate only in a few caudal segments (Fig. 2D, G). Pore formula normal: 5, 7, 9, 10, 12, 13, 15–19. Ozopores small, round, opening flush dorsally near caudal corner of poriferous paraterga. Stricture between pro- and metazonae wide, shallow. Limbus very finely microspiculate. All spiracles usual, simple. Pleurosternal carinae traceable as very faint lines on most segments (Fig. 2B, D). Epiproct short, conical, flattened dorsoventrally. Hypoproct semi-circular, setae strongly separated and borne on minute knobs.

Sterna wide, unmodified, setose. Legs rather long and slender, ca 1.2–1.3 (♂) or 1.0–1.1 (♀) times as long as midbody height; in length, tarsus > femur > prefemur > coxa = postfemur = tibia, the latter with a particularly long, tactile seta apicodorsally. Tarsal brushes absent.

**Figure 3.**
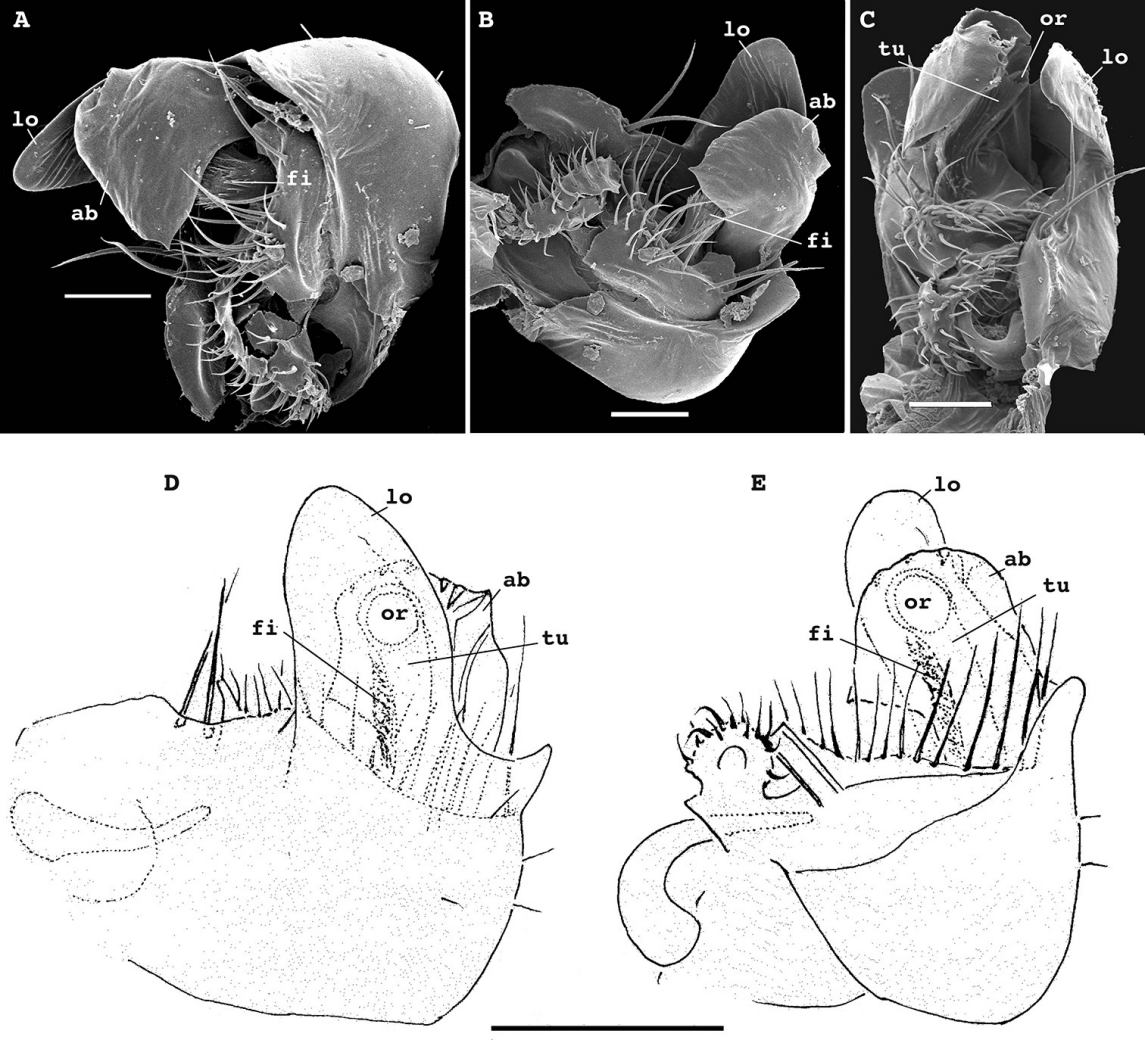
*Physetoparia complexa* sp. nov., gonopods of ♂ paratypes **A, B** left gonopod, lateral and ventrolateral views, respectively **C** right gonopod, ventrocaudal view **D, E** right gonopod, lateral and mesal views, respectively. Abbreviations: **lo** distolateral lobe of coxa, **ab** apicomesal branch/outgrowth of telopodite, **tu** tube/solenomere between **lo** and **ab** with a broad orifice (**or**) and a field of filaments (**fi**) at base.Scale bars: 0.05 mm.

Gonopods (Fig. 3) with large, subglobose, barely setose coxae, fused medially at base, each coxa carrying a very prominent, rounded, distolateral lobe (lo) and two very strong setae near place of fusion. Telopodites very clearly exposed, but strongly protected by lo, bases only a little concealed inside a shallow gonocoel. Telopodites only slightly shorter than lo, each with only a single, large, subsecuriform, lobe-shaped, apicomesal branch/outgrowth (ab) showing a microdentate apical margin, a peculiar tube (tu) with a large orifice (or), and a field of fimbriae at base of tu, both hidden between lo and ap; tu apparently functioning as a solenomere.

##### Remarks.

This new species shows several clear-cut apomorphies in gonopodal characters (see Diagnosis above), but on balance it fits quite well the scope of *Physetoparia* as outlined by Golovatch et al. (2018). Especially distinct similarities concern the sole congener that has a marked apicolateral outgrowth/lobe on the gonopodal coxa to protect a likewise well exposed telopodite: *P. villiersi* (Schubart, 1955). However, the gonotelopodite in the latter species is tripartite, including a finger-shaped solenomere, while the coxal lobe is much smaller and less conspicuous (Schubart 1955). In addition, both these species compared come from the same area, the Nimba Mountains which are shared by Liberia, Guinea and Côte d’Ivoire.

#### Bactrodesmus

Taxon classificationAnimaliaPolydesmidaFuhrmannodesmidae

Cook, 1896

90EC05A4-12FF-50E0-8666-67F1A8E59A01

##### Type species.

*Bactrodesmus claviger* Cook, 1896, by subsequent monotypy, Liberia.

As reiterated recently (Golovatch et al. 2018), this genus was first proposed as a *nomen nudum* (Cook 1896a), but then properly typified (Cook 1896b). The sole useful information contained in the original description of *B. claviger*, which was accompanied by no illustrations, concerns its small size (7 mm long, 1 mm wide), typically micropolydesmid facies (small paraterga, large and clubbed tergal setae arranged in three transverse rows etc.), strongly enlarged gonocoxae that fully conceal the telopodites and, above all, ♂ legs 2, especially their tibiae, greatly enlarged compared to others (Cook 1896b). No number of body segments has been given.

Below we put on record a new *Bactrodesmus* coming from the Guinean portion of the Nimba Mountains. This allows us to unequivocally clarify the identity of the genus and provide a new diagnosis.

##### Diagnosis.

At least ♂ tibiae 2, as well as both gonopodal coxae and gonocoel hypertrophied, telopodites being strongly sunken and their distal outgrowths remaining nearly fully concealed inside gonocoel. Only one prominent, basal fold/branch (bb = sp) present, albeit fully concealed as well; a simple and short solenomere branch (sl) protected by bb mesally and by a clearly 2-segmented lateral part laterally.

##### Remark.

This genus is presumably among the most advanced representatives of Afrotropical Trichopolydesmidae in showing several autapomorphies.

#### Bactrodesmus grandis sp. nov.

Taxon classificationAnimaliaPolydesmidaFuhrmannodesmidae

4BA61427-9DC9-55A1-876B-EA45025DCFD7

http://zoobank.org/AF5E4B4D-7A9B-426D-A87D-16EC8FFB61BD

Figs 1B, C , 4 , 5

##### Type material.

***Holotype*** ♂ (MRAC 22843), Guinea, Nimba Mountains, near cave 2, Serengbara, camp 3, ca 1035 m a.s.l., litter, 2.V.2019, A. Henrard, D. VandenSpiegel, C. Allard et al. leg. (Nimba 2019-41). ***Paratypes***: 1 ♀ (MRAC 22844), same locality, together with holotype: 2 ♂, 1 ♀ (MRAC 22845), 2 ♂ (MRAC 22862), 1 ♂ (SEM, MRAC 22846), 1 ♂ (ZMUM Rd 4628), same locality, forest; ca 975 m a.s.l., 2.V.2019, A. Henrard, D. VandenSpiegel, C. Allard et al. leg. (Nimba 2019-49).

##### Diagnosis.

Differs from both other species of the genus by ♂ legs 1–3 being clearly enlarged and modified, vs. ♂ legs 2 or 2 and 3, from *B. bicornis* also by three (vs. two) transverse rows of tergal setae and the collum which is narrower than the head, from *B. claviger* by the considerably larger body.

##### Name.

To emphasize the relatively large body and clearly enlarged ♂ legs 1–3; adjective.

##### Description.

Length ca 8 (♂, including holotype) or 9 mm (♀), width of midbody pro- and metazonae 1.0 and 1.3 mm (♂, including holotype) or 1.2 and 1.5 mm (♀), respectively. Coloration in alcohol marbled light brown to reddish brown, venter and legs usually lighter, light grey-brown to nearly pallid (Fig. 1B, C).

**Figure 4.**
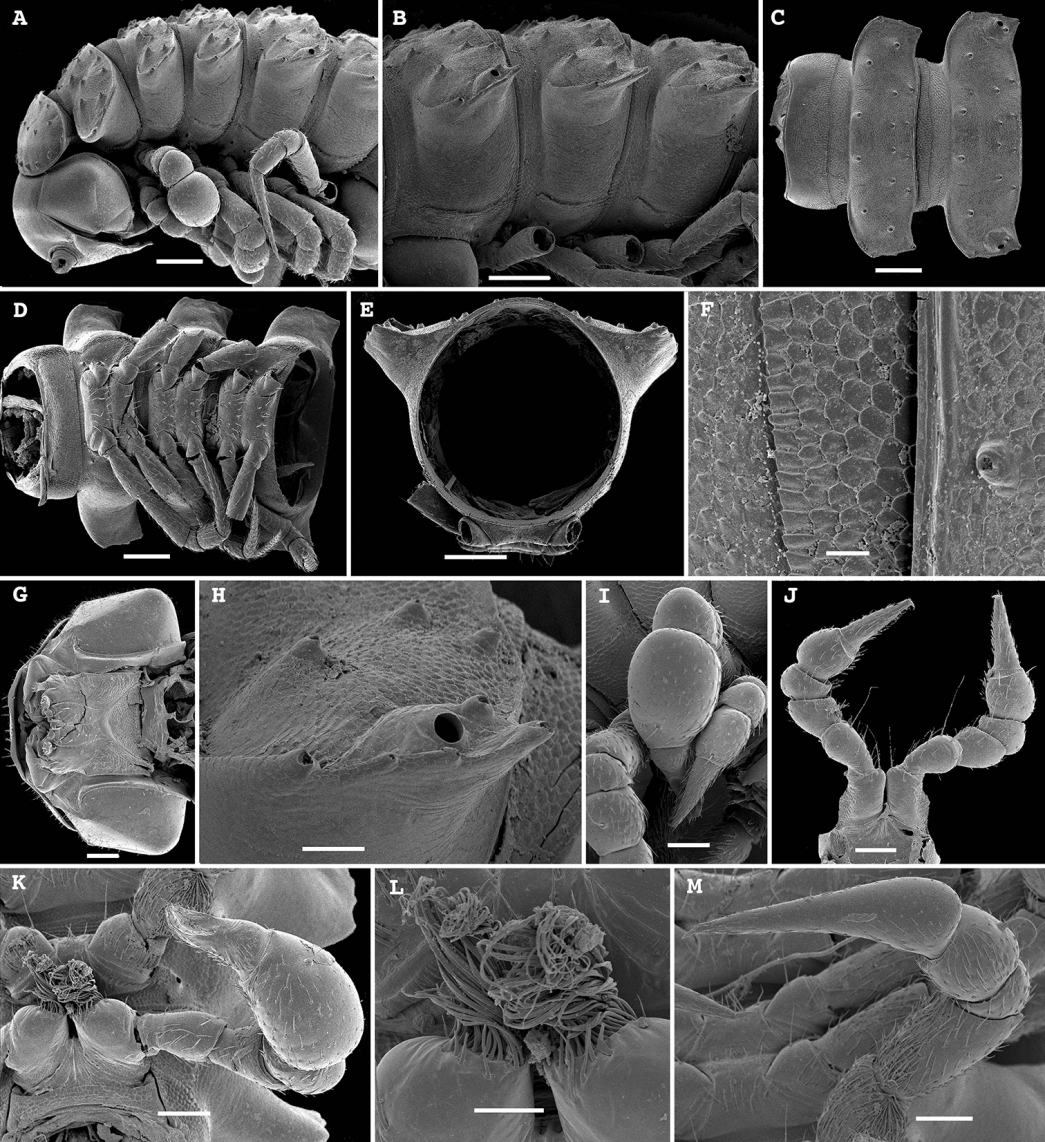
*Bactrodesmus grandis* sp. nov., SEM micrographs of a ♂ paratype **A** anterior part of body, lateral view **B–D** midbody segments, lateral, dorsal and ventral views, respectively **E** cross-section of a midbody segment, caudal view **F** fine tergal structure, dorsal view **G** head, ventral view **H** midbody paratergum, lateral view **I** from right to left, legs 1–3 in situ, lateral view **J** leg-pair 1, oral view **K** leg 2 and base of leg 3, frontoventral view **L** coxae 2, subventral view **M** leg 3 and bases of several following legs, frontoventral view. Scale bars: 0.2 mm (**A–E**), 0.1 mm (**G, I–K, M**), 0.05 mm (**I, L**), 0.02 mm (**F**).

Body with 20 segments in both sexes. Tegument very delicately micro-alveolate, mainly slightly shining. Head densely micropilose, devoid of epicranial modifications, but genae roundly squarish and very strongly swollen laterally; gnathochilarium without modifications (Fig. 4G). Interantennal isthmus 1.8 times diameter of antennal socket. Antennae long and strongly clavate, reaching back past segment 3 (♂) when stretched dorsally. In length, antennomere 3 = 6 > 5 > 2 = 4 > 7 > 1; antennomere 6 the largest, antennomeres 5 and 6 each with a distinct, round, distodorsal field of minute sensilla. In width, collum < segments 2 and 3 < head = 4 < 5–16; thereafter body gradually tapering towards telson. Collum ellipsoid, transversely oval, like all following metaterga with three transverse, regular rows of setae. Tergal setae largely abraded, medium-sized, each ca 1/4–1/5 as long as metatergum, bacilliform and longitudinally ribbed, set on minute knobs, growing slightly longer toward telson, 3–4 additional setae present at lateral margin of paraterga (Fig. 4A–E, H), always 3+3 in each row on postcollum metaterga. Dorsal surface of metaterga nearly smooth, regularly convex. Paraterga medium-sized, set at around upper 1/3 of metazonae (Fig. 4A–C, E, H), visible starting with collum, often slightly upturned caudally, faintly, but regularly rounded and bordered, lateral incisions absent, with minute setigerous knobs present in their stead, including ones located at caudal corners. Paraterga 2 slightly enlarged, more strongly declined and broadly rounded compared to following ones (Fig. 4A). Starting with paraterga 5 or 6, caudal corner increasingly sharp and drawn back past rear tergal margin (Fig. 4A–C, H). Pore formula normal: 5, 7, 9, 10, 12, 13, 15–19. Ozopores small, round, opening flush dorsally near caudal corner of poriferous paraterga. Stricture between pro- and metazonae wide, shallow. Limbus very finely microspiculate. Spiracles very small, located on short cones (Fig. 4K). Pleurosternal carinae traceable as very faint ridges or lines on most segments (Fig. 4A, B). Epiproct short, conical, flattened dorsoventrally. Hypoproct semi-circular, setae strongly separated and borne on minute knobs.

Sterna wide, unmodified, setose. Legs rather long and slender, ca 1.3–1.4 (♂) or 1.1–1.2 times (♀) as long as midbody height; in length, tarsus > femur > prefemur > coxa = postfemur = tibia. Tarsal brushes present only on ♂ legs 1 and 2; ♂ legs 1–3 conspicuously enlarged (Fig. 4I): legs 1 (Fig. 4J) with increasingly inflated pretarsal podomeres; legs 2 (Fig. 4K, L) with each coxa caudally supplied with what seems to be a gland whose wide orifice is surrounded by a whorl of setae while the interior carries bundles of abundant, very long, sharp, distally entangled filaments; tibiae 2 particularly strongly swollen, while tarsi 2 somewhat shortened, dorsally flattened and spoon-shaped; legs 3 (Fig. 4K, M) resembling legs 1, but their prefemora and femora especially densely setose ventrally.

**Figure 5.**
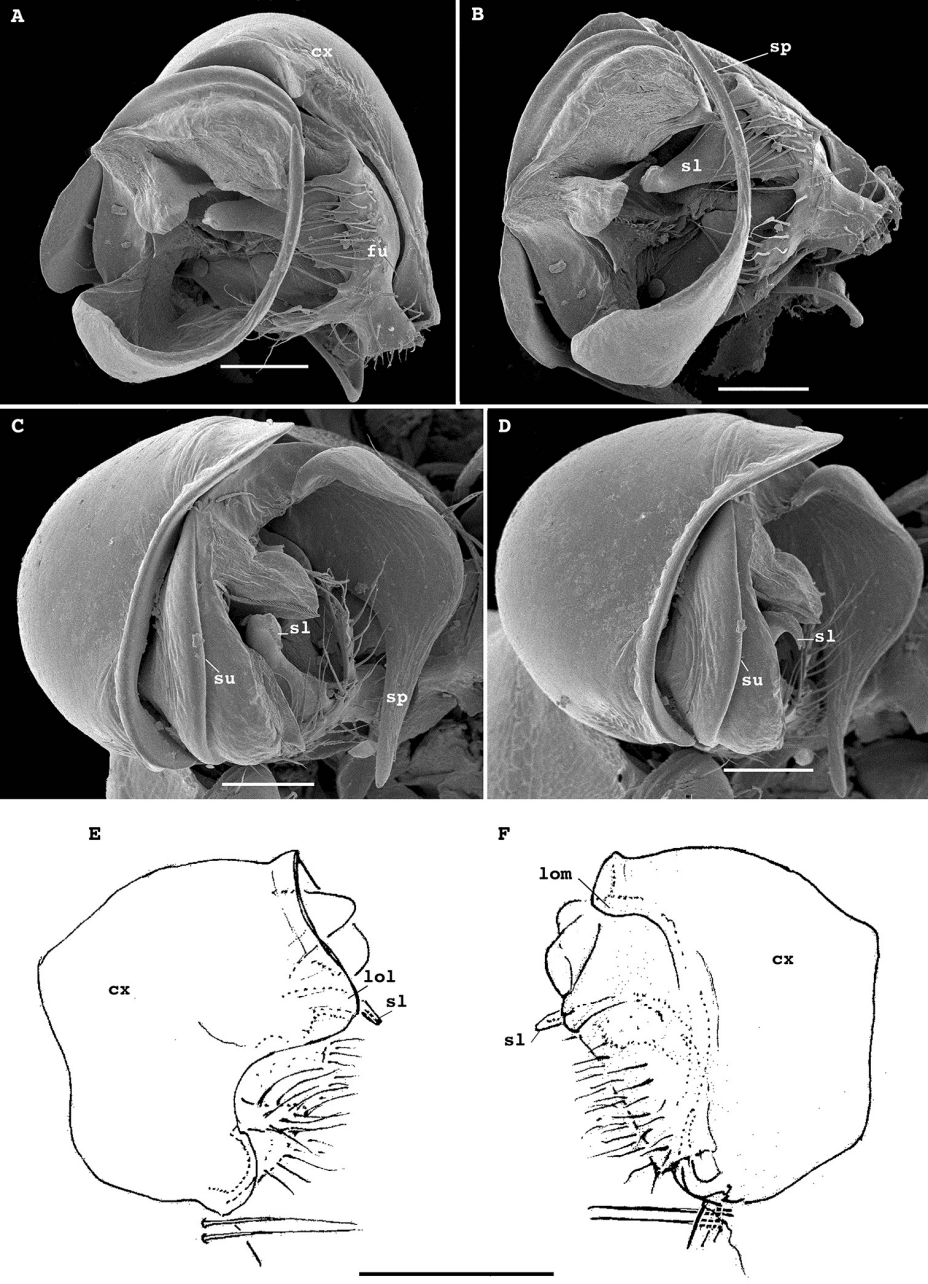
*Bactrodesmus grandis* sp. nov., gonopods of ♂ paratypes **A, B** left gonopod, subventral and ventromesal views, respectively **C, D** right gonopod, ventrolateral and ventral views, respectively **E, F** right gonopod, lateral and mesal views, respectively. Abbreviations: **cx** coxa, **lol** distolateral lobe of coxa, **lom** distomesal lobe of coxa, **fu** basal funnel of telopodite, **sl** solenomere, **sp** spine, **su** parabasal sulcus on telopodite. Scale bars: 0.1 mm.

Gonopods (Fig. 5) complex, with particularly strongly enlarged, globose and nearly smooth coxae (cx), both forming a very deep gonocoel, both clearly rimmed apically and with 2+2 especially strong setae mediobasally near place of coxal fusion; one small rounded lobe each present on cx distolaterally (lol) and distomesally (lom); cannulae relatively small, as usual. Telopodites deeply sunken inside gonocoel, very poorly exposed beyond it, each starting with a setose funnel-shaped part (fu) marking the orifice for the cannula to enter and the beginning of a seminal groove, the latter quickly passing onto a short, stout, slightly curved, distad attenuating solenomere (sl) branch fully concealed inside gonocoel; basal part of telopodite extended mesally along fu into a distinct fold turning apically into a long, gently and regularly curved, laterad directed spine (sp); lateral part of telopodite divided distally by a clear-cut suture (su) into two sections, both being simple and stout slabs, but distal one bearing a meso-central membranous sac to protect sl tip.

##### Remarks.

The size, external structures and gonopodal conformation of *B. grandis* sp. nov. match closely those as described and depicted for *B. bicornis* by Demange and Mauriès (1975). The latter species is 8.0 mm long and 1.5 mm wide. Its hypertrophied gonopodal coxa is likewise nearly smooth and shows two small distal lobes, lol and lom. The short spiniform solenomere (sl), the long mesobasal spine (sp) and the two-segmented lateral part of the gonotelopodite look much like, and are located similarly in *B. grandis* sp. nov. Unfortunately, even though the gonopodal structure of *B. claviger* remains unknown, the genus *Bactrodesmus* can presently be redefined (see above).

#### Hemisphaeroparia parvula

Taxon classificationAnimaliaPolydesmidaFuhrmannodesmidae

(Porat, 1894) comb. nov.

B99501C0-A1AE-5CB3-AD52-7185939838E0

Fig. 1D

Polydesmus parvulus Porat, 1894: 31 (original description).

##### Type material.

**Syntypes** 2 ♀ (NHRM-GULI000069465), Kamerun, Yngve Sjöstedt leg.

##### Remarks.

Porat (1894) described this species, based on two syntypes deriving from an unspecified locality in Cameroon. We have revised both syntypes and found them to be adult females, one incomplete, the other one complete and with 20 segments (Fig. 1D). Since Cameroon appears to support solely species of the trichopolydesmid genus *Hemisphaeroparia* (24 at the moment), we tentatively transfer the above species to *Hemisphaeroparia*, comb. nov., even though the spiracles located next to coxa 1 or 2 are not enlarged (Fig. 1D). Characteristically enlarged spiracles 1 appear to be restricted to far from all species of *Hemisphaeroparia* (see below under *H. spiniger*). We doubt though that the identity of this enigmatic species will ever be properly established, as superficially the females of most species of Trichopolydesmidae look very much alike. Only the everted vulvae of one of the syntypes might be helpful in the future, but first their comparative study must be accomplished.

#### Hemisphaeroparia falcata

Taxon classificationAnimaliaPolydesmidaFuhrmannodesmidae

Golovatch, Nzoko Fiemapong, Tamesse, Mauriès & VandenSpiegel, 2018

C43704D8-F036-54C1-ACD8-9E48431D7918

Figs 6 , 7

Hemisphaeroparia falcata Golovatch et al., 2018: 84 (original description).

##### New material.

1 ♂ (MRAC 22847), 1 ♂ (SEM, MRAC 22848), Cameroon, Center Region, Mafou and Afamba Division, Mfou, cocoa plantation, 3°48’49.6”N, 11°40’49.6”E, 24.VII.2019, A.R. Nzoko Fiemapong leg.

##### Remarks.

**Figure 6.**
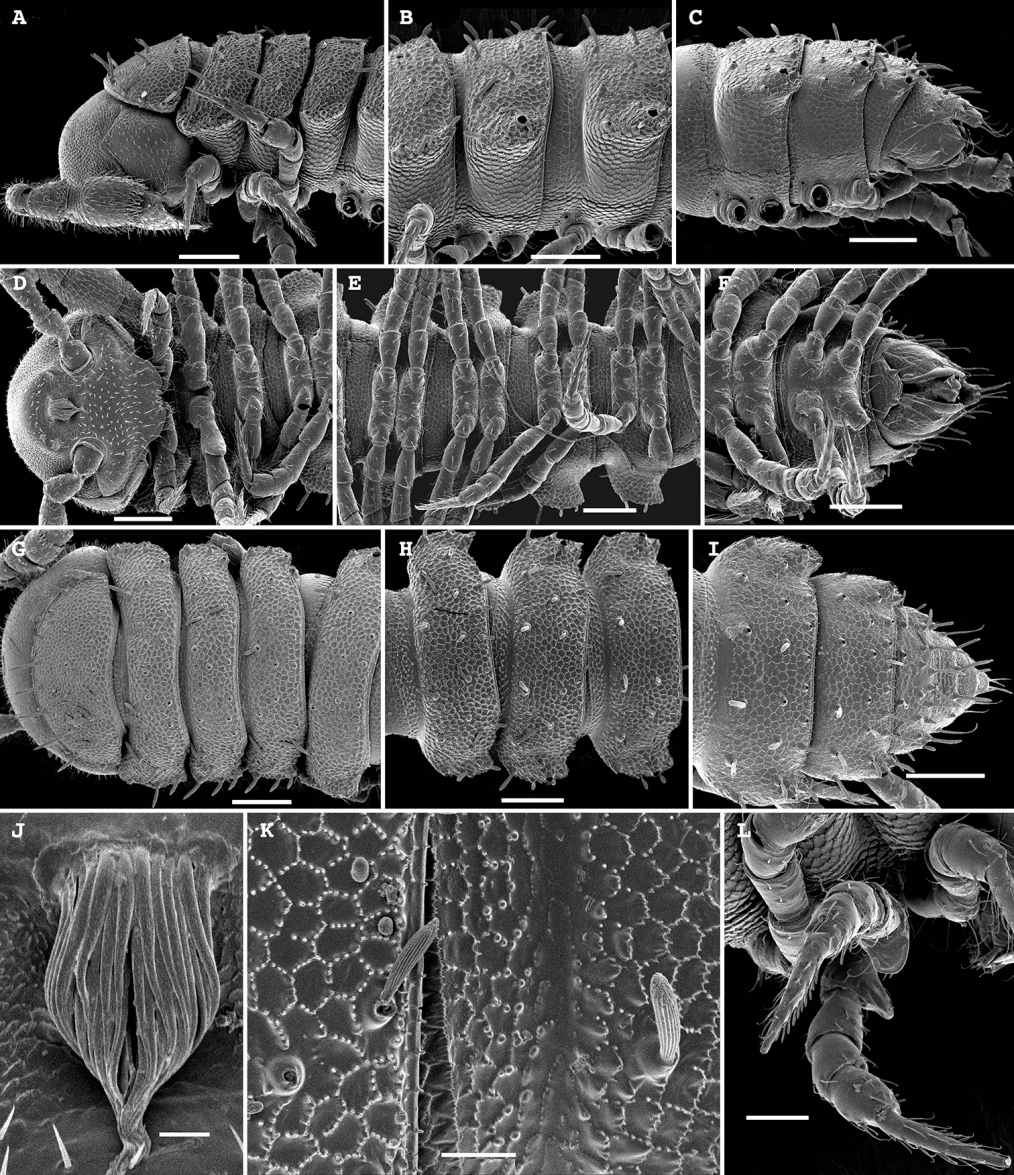
*Hemisphaeroparia falcata* Golovatch, Nzoko Fiemapong, Tamesse, Mauriès & VandenSpiegel, 2018, SEM micrographs of ♂ from Mfou **A, D, G** anterior part of body, lateral, ventral and dorsal views, respectively **B, E, H** midbody segments, lateral, ventral and dorsal views, respectively **C, F, I** posterior part of body, lateral, ventral and dorsal views, respectively **J** epicranial bundles of filaments, dorsal view **K** fine tergal structure with setae, dorsal view **L** anterior legs with a triangular ventral process on prefemur 1, lateral view. Scale bars: 0.1 mm (**A–I**), 0.05 mm (**L**), 0.02 mm (**K**), 0.01 mm (**J**).

**Figure 7.**
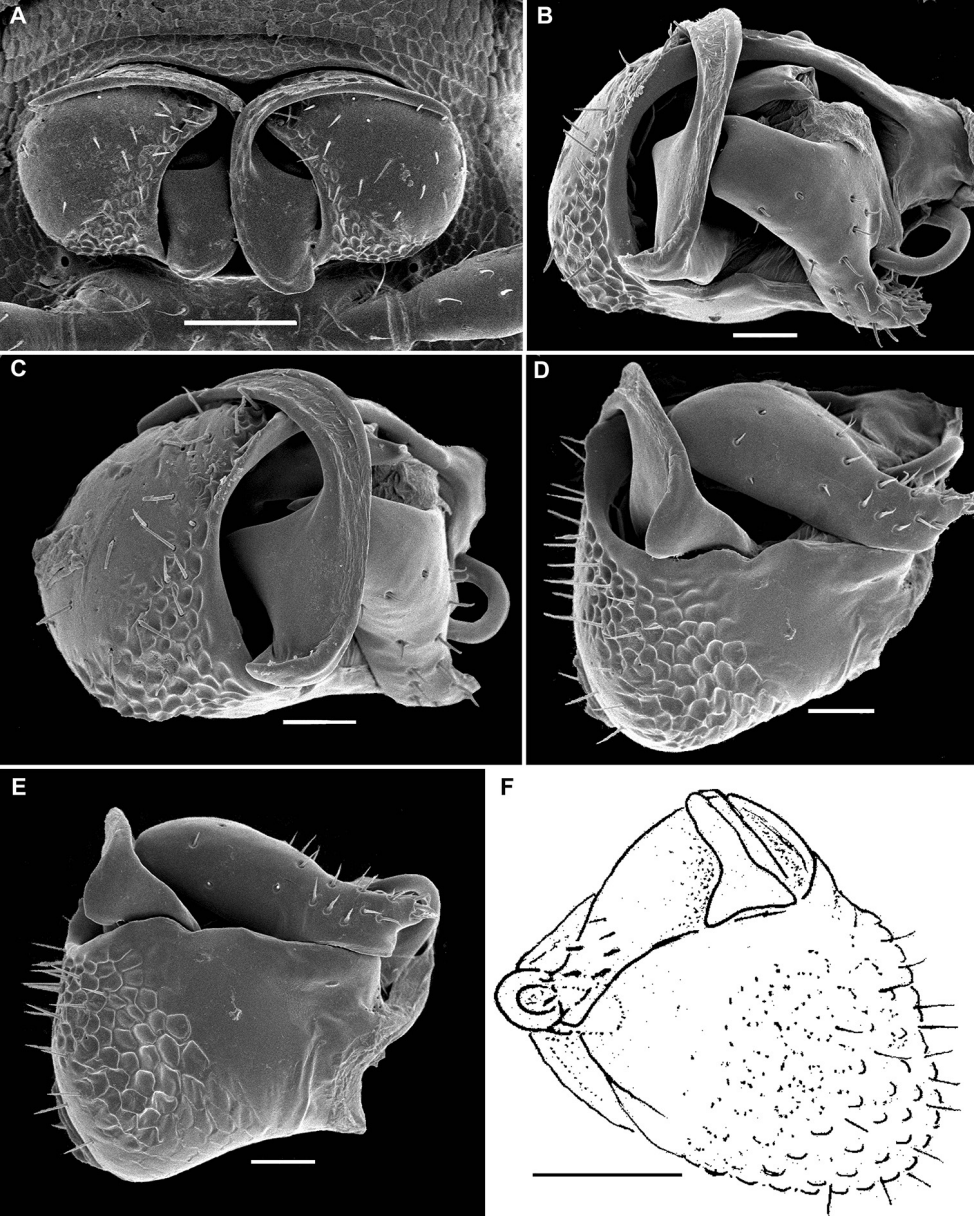
*Hemisphaeroparia falcata* Golovatch, Nzoko Fiemapong, Tamesse, Mauriès & VandenSpiegel, 2018, gonopods of ♂♂ from Mfou **A** both gonopods in situ, ventral view **B–E** left gonopod in various views **F** right gonopod, caudal view. Scale bars: 0.05 mm (**A, F**), 0.02 mm (**B–E**).

The new samples fully agree with the original description (Golovatch et al. 2018) and are again illustrated not only to confirm the species’ identity (Figs 6, 7), including the unique, conspicuous, epicranial bundles of long filaments on the ♂ head (Fig. 6D, J), but also to note the presence of a marked ventrobasal process on each ♂ prefemur 1 (Fig. 6L), which is much like the one observed in *H. avis* sp. nov.

The new locality, Mfou, lies quite close to the type one, Awae, both in the Central Region of Cameroon. Because Awae represents a native woodland habitat, *H. falcata* might have been introduced to the cocoa plantation at Mfou.

#### Hemisphaeroparia spiniger

Taxon classificationAnimaliaPolydesmidaFuhrmannodesmidae

Golovatch, Nzoko Fiemapong, Tamesse, Mauriès & VandenSpiegel, 2018

ACCB1AA0-857F-5647-95A3-F4AEEA9AD65E

Figs 8 , 9

Hemisphaeroparia spiniger Golovatch et al., 2018: 64 (original description).

##### New material.

1 ♂ (MRAC 22860), 1 ♂ (SEM, MRAC 22861), Cameroon, Center Region, Mafou and Afamba Division, Mfou, cocoa plantation, 3°48’49.6”N, 11°40’49.6”E, 24.VII.2019, A.R. Nzoko Fiemapong leg.

##### Remarks.

**Figure 8.**
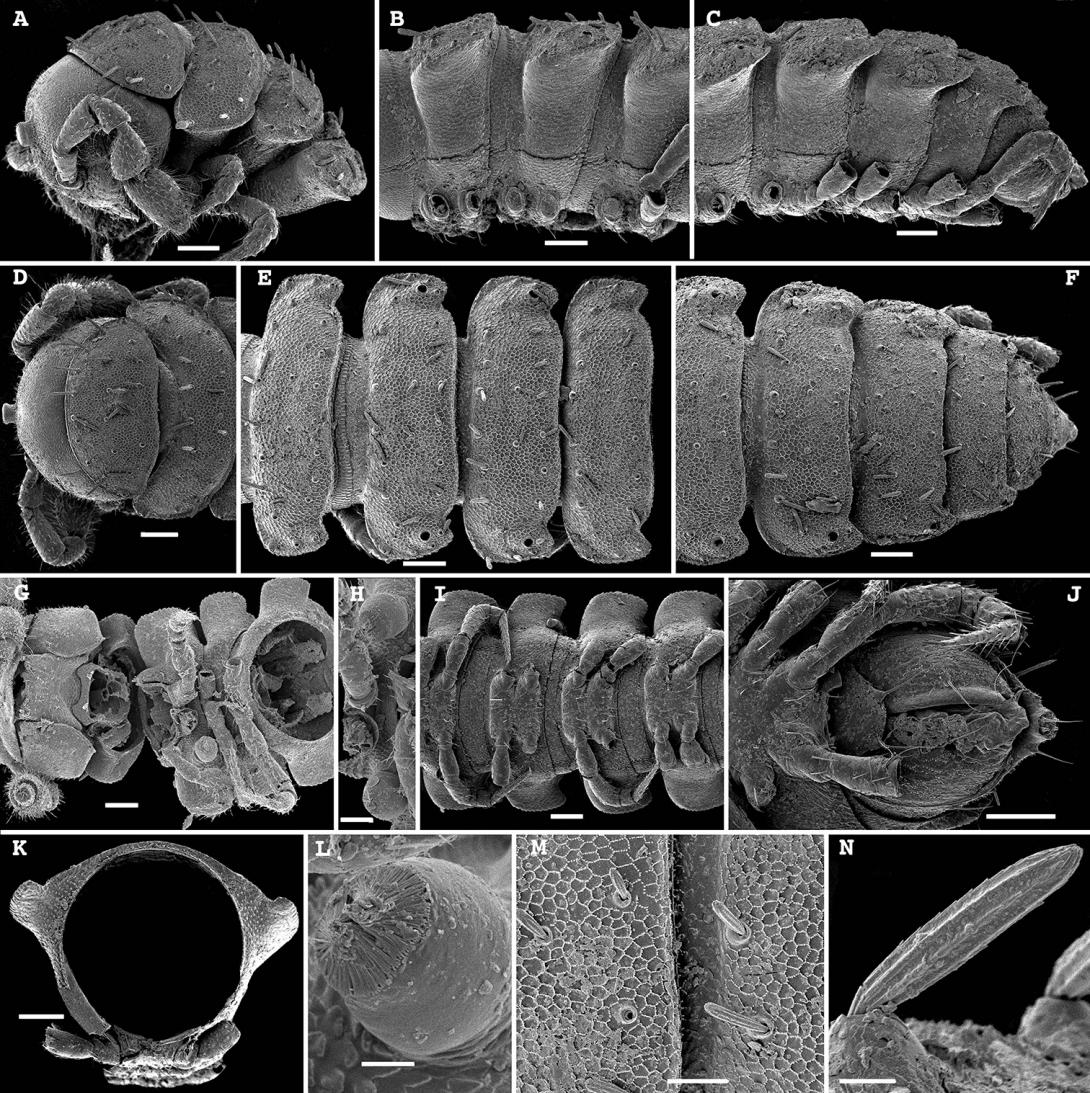
*Hemisphaeroparia spiniger* Golovatch, Nzoko Fiemapong, Tamesse, Mauriès & VandenSpiegel, 2018, SEM micrographs of ♂ from Mfou **A, D, G** anterior part of body, lateral, dorsal and ventral views, respectively **B, E, I** midbody segments, lateral, dorsal and ventral views, respectively **C, F, J** posterior part of body, lateral, dorsal and ventral views, respectively **H, L** enlarged spiracles near coxae 2, ventral view **K** cross-section of a midbody segment, caudal view **M** fine tergal structure with setae, dorsal view **N** tergal seta, enlarged. Scale bars: 0.1 mm (**A–G, I–K**), 0.05 mm (**H, M**), 0.02 mm (**L**), 0.01 mm (**N**).

**Figure 9.**
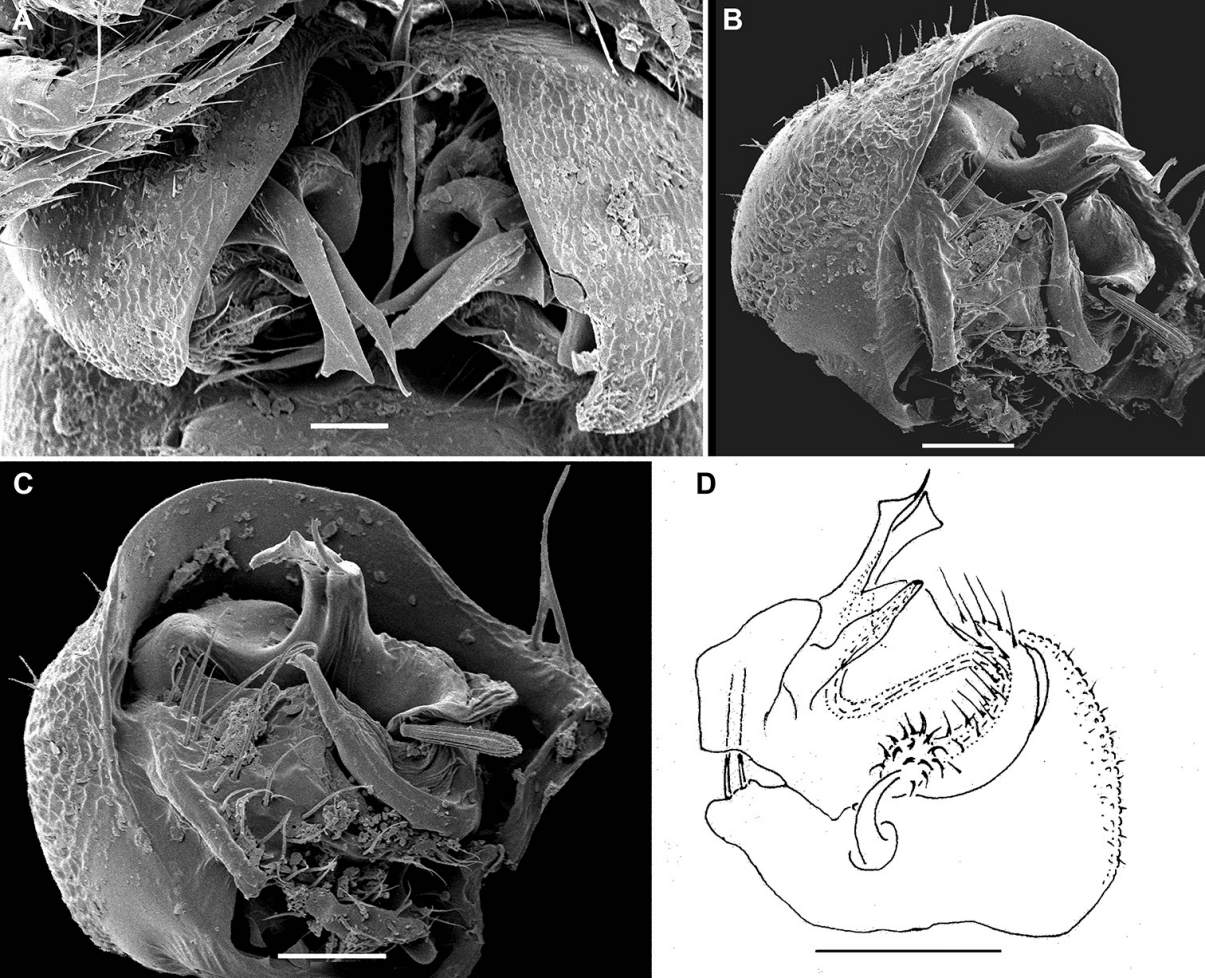
*Hemisphaeroparia spiniger* Golovatch, Nzoko Fiemapong, Tamesse, Mauriès & VandenSpiegel, 2018, gonopods of ♂♂ from Mfou **A** both gonopods in situ, ventral view **B, C** right gonopod, caudolateral and subcaudal views, respectively **D** left gonopod, mesal view. Scale bars: 0.1 mm (**D**), 0.05 mm (**A–C**).

The new samples fully agree with the original description (Golovatch et al. 2018) and are again illustrated to confirm the species’ identity (Figs 8, 9), including the remarkably enlarged spiracles 1.

The new locality, Mfou, lies quite close to the type one, campus of University Yaounde 1, both in the Central Region of Cameroon. Moreover, because both known localities/habitats represent artificial palm or cocoa plantations, *H. spiniger* could have been introduced there from some native woodlands still to be revealed or already vanished.

#### Hemisphaeroparia longibrachiata sp. nov.

Taxon classificationAnimaliaPolydesmidaFuhrmannodesmidae

CA607B8F-432D-589D-B755-CB4122C71341

http://zoobank.org/2B3015B9-3869-471D-B387-C5B3B840FEB4

Figs 1E , 10 , 11

##### Type material.

***Holotype*** ♂ (MRAC 22857), Cameroon, West Region, Haut-Nkam Division; sacred forest, 5,313712N, 10,250323E, 28.V.2019, A.R. Nzoko Fiemapong leg.

***Paratypes***, 2 ♂, 2 ♀, 1 ♀ fragment (MRAC 22858), 1 ♂ (SEM, MRAC 22859), 1 ♂ (UY1), 1 ♂ (ZMUM Rd 4629), same locality, together with holotype.

##### Diagnosis.

Differs from all other species of the genus by the presence of only 19 segments in both sexes, coupled with a distinct, central, setose pit with two paramedian pores at the bottom in the ♂ epicranium, and the particularly long, falcate, fully exposed branch/process ab on the gonopodal telopodite.

##### Name.

To emphasize the particularly long branch/process ab on the gonopodal telopodite; adjective in feminine gender.

##### Description.

Length of holotype ca 4 mm (♂), width of midbody pro- and metazonae 0.3 and 0.5 mm (♂), respectively. Length of paratypes 4–5 mm, width of midbody pro- and metazonae 0.3–0.4 and 0.5–0.6 mm (♂, ♀), respectively. Coloration in alcohol faintly marbled, light brown to brown, venter and legs light grey-brown (Fig. 1E).

**Figure 10.**
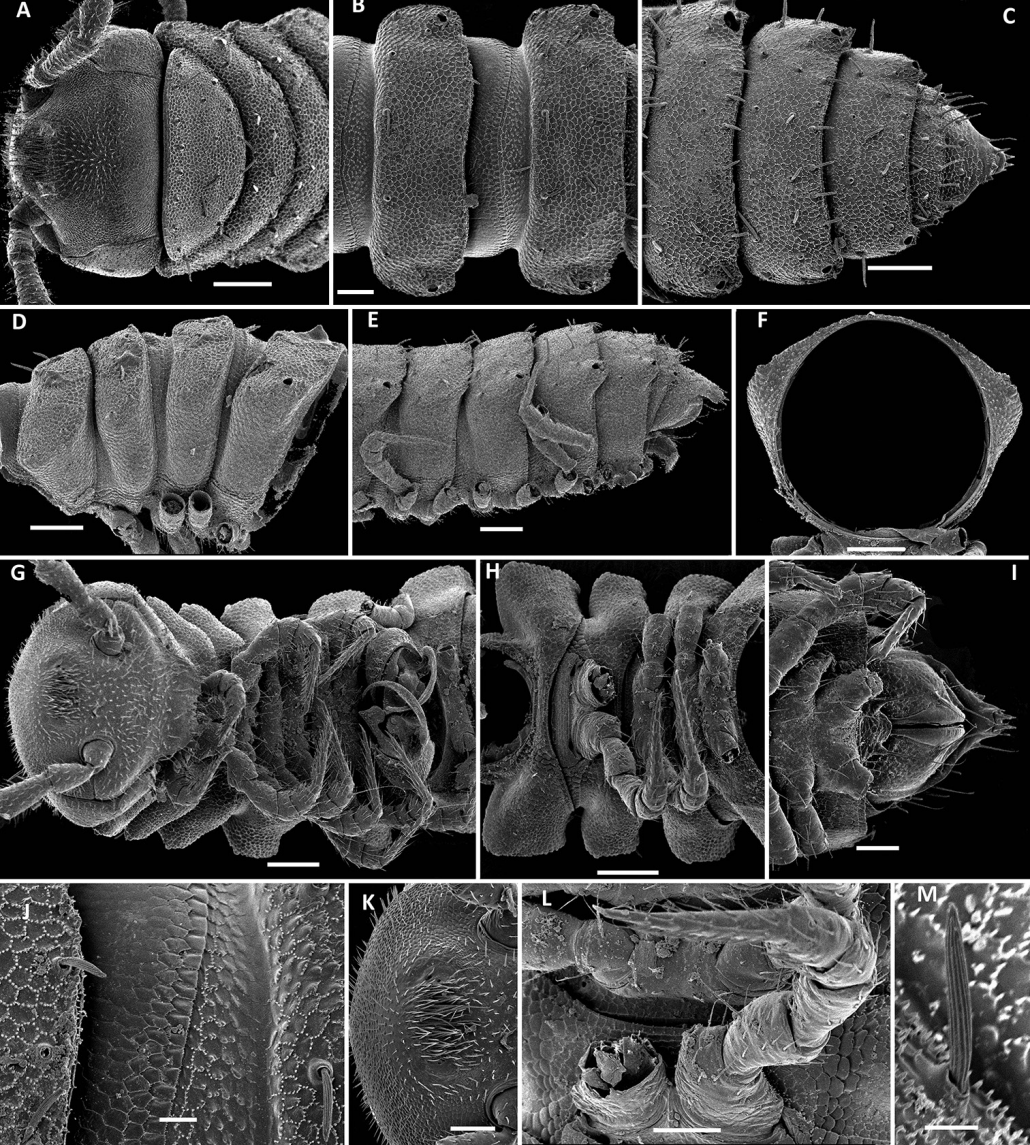
*Hemisphaeroparia longibrachiata* sp. nov., SEM micrographs of a ♂ paratype **A, G** anterior part of body, dorsal and ventral views, respectively **B, D, H** midbody segments, dorsal, lateral and ventral views, respectively **C, E, I** posterior part of body, dorsal, lateral and ventral views, respectively **F** cross-section of a midbody segment, caudal view **J** fine tergal structure with setae, dorsal view **K** epicranial pit, dorsal view **L** leg 2 with gonopore on coxa **M** limbus and tergal seta, enlarged. Scale bars: 0.1 mm (**A–G, I–K**), 0.05 mm (**H, M**), 0.02 mm (**L**), 0.01 mm (**N**)

Body with 19 segments in both sexes. Tegument very delicately micro-alveolate, mainly slightly shining. Head very densely micropilose, ♂ epicranium slightly elevated and supplied with a very distinct, central, oval, densely setose pit with two paramedian pores (Fig. 10G, K). Interantennal isthmus almost three times diameter of antennal socket. Antennae long and strongly clavate, reaching back past segment 4 (♂) or 3 (♀) when stretched dorsally. In length, antennomere 3 = 6 > 5 > 2 = 4 > 7 > 1; antennomere 6 the largest, antennomeres 5 and 6 each with a distinct, round, distodorsal field of sensilla. In width, segments 5–15 >2 > head = segments 3 and 4 > collum; body gradually tapering towards telson on segments 16–19. Collum ellipsoid, transversely oval, like all following metaterga with three transverse, regular rows of setae; anterior row composed of somewhat longer setae. Tergal setae medium-sized, each ca 1/5 as long as metatergum, bacilliform and longitudinally ribbed (Fig. 10A–E, I, M), always 3+3 in each row on postcollum metaterga; 2–3 additional setae at lateral margin of paraterga. Dorsum invariably regularly convex. Paraterga medium-sized, set at around upper 1/3 of metazonae (Fig. 10D–F), visible starting with collum, regularly rounded, lateral incisions absent. Caudal corner of paraterga mostly rounded, drawn back past rear tergal margin only on segments 16 and 17 (Fig. 10C, E). Pore formula normal: 5, 7, 9, 10, 12, 13, 15–18. Ozopores small, round, opening flush dorsally near caudal corner of poriferous paraterga. Stricture between pro- and metazonae wide, shallow. Limbus very finely microspiculate. Spiracles very small, as usual. Pleurosternal carinae traceable as very faint ridges or lines on most segments (Fig. 10D, E). Epiproct short, conical, flattened dorsoventrally. Hypoproct semi-circular, setae strongly separated and borne on minute knobs.

Sterna wide, unmodified, setose. Legs rather long and slender, ca 1.2–1.3 (♂) or 1.0–1.1 (♀) times as long as midbody height; in length, tarsus > femur > coxa = prefemur = postfemur = tibia, the latter with a particularly long, tactile seta apicodorsally. Tarsal brushes absent.

**Figure 11.**
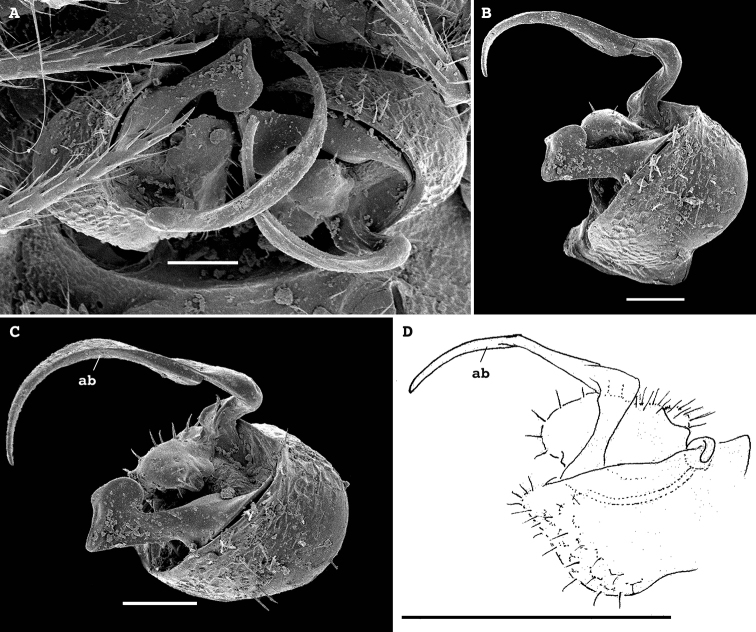
*Hemisphaeroparia longibrachiata* sp. nov., gonopods of ♂ paratypes **A** both gonopods in situ, ventral view **B, C** left gonopod, caudolateral and subcaudal views, respectively **D** right gonopod, mesal view. Abbreviation: **ab** apical branch. Scale bars: 0.1 mm (**D**), 0.05 mm (**A–C**).

Gonopods (Fig. 11) with large, subglobose, clearly exposed, alveolate coxae, these rather densely setose nearly throughout, fused medially at base, each carrying two very long setae near place of fusion. Telopodites largely well exposed beyond a moderately deep gonocoel, each with two low bulges basal to anterior branch (ab), the latter extremely long, slightly coiled in basal third, falcate, gradually attenuating towards a narrowly rounded tip. No solenomere discernible at base of ab.

#### Hemisphaeroparia avis sp. nov.

Taxon classificationAnimaliaPolydesmidaFuhrmannodesmidae

B4ECECAC-750E-5CA8-8CD2-75E63C58BCF0

http://zoobank.org/3BEC5271-1547-4F69-9757-7629D354F257

Figs 1F , 12 , 13

##### Type material.

***Holotype*** ♂ (MRAC 22853), Cameroon, Center Region, Mafou and Afamba Division, Mfou, cocoa plantation, 3°48’49.6”N, 11°40’49.6”E, 24.VII.2019, A.R. Nzoko Fiemapong leg. ***Paratypes***: 3 ♂, 12 ♀, 2 subadult ♀ (many fragmented) (MRAC 22854), 12 ♂ (MRAC 22855), 1 ♂ (SEM, MRAC 22856), 1 ♂, 1 ♀ (ZMUM Rd 4630), 1 ♂ (UY1), same locality, together with holotype.

**Figure 12.**
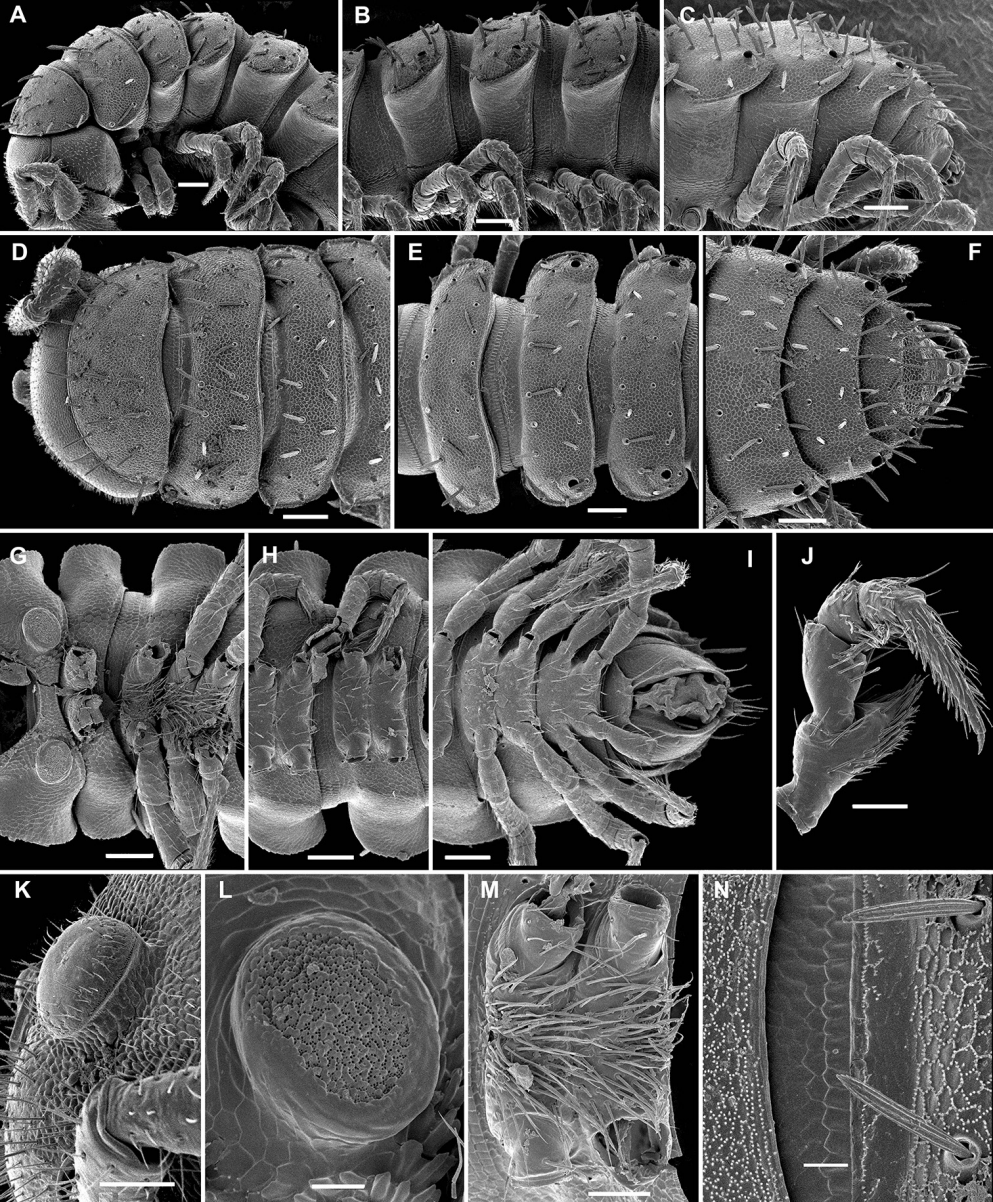
*Hemisphaeroparia avis* sp. nov., SEM micrographs of a ♂ paratype **A, D, G** anterior part of body, lateral, dorsal and ventral views, respectively **B, E, H** midbody segments, lateral, dorsal and ventral views, respectively **C, F, I** posterior part of body, lateral, dorsal and ventral views, respectively **J** telopodite 1 with a prominent process in prefemur **K, L** epicranial tubercle **M** densely setose sterna between coxae 2 and 3 **N** fine tergal structure with limbus and setae, dorsal view. Scale bars: 0.1 mm (**A–I**), 0.05 mm (**J, K, M**), 0.02 mm (**L, N**).

**Figure 13.**
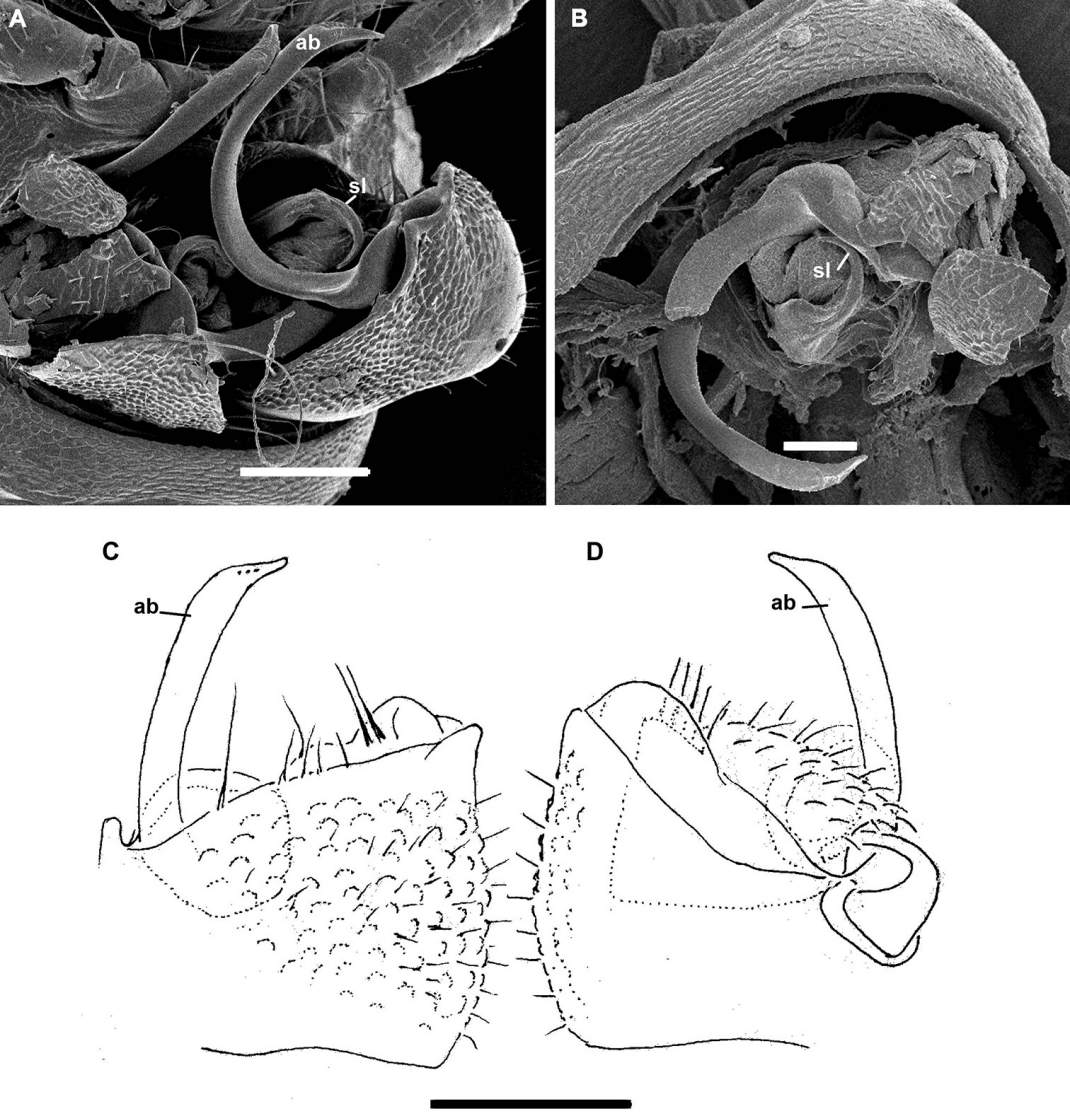
*Hemisphaeroparia avis* sp. nov., gonopods of ♂ paratypes **A** both gonopods in situ, ventral view **B–D** right gonopod, ventrocaudal, lateral and mesal views, respectively. Abbreviations: **ab** apical branch, **sl** solenomere. Scale bars: 0.1 mm (**A, C, D**), 0.05 mm (**B**).

**Diagnosis.** Differs from all other species of the genus by the presence of a boletiform epicranial tubercle (♂) (Fig. 12D, K), coupled with the unusually large, disc-shaped spiracles next to coxae 1 or 2 (Fig. 12G, L), the strong, setose, subtriangular, distoventral process on ♂ prefemur (Fig. 12J), the densely setose sterna between ♂ coxae 2 and 3 (Fig. 12M), and the sole prominent, clearly exposed process (ab) with a bird’s beak-shaped tip on the gonopodal telopodite (Fig. 13).

**Name.** From Latin *avis* (= bird), to emphasize the bird’s beak-shaped tip of the sole process (ab) of the gonopodal telopodite; noun in apposition.

**Description.** Length of holotype ca 4.5 mm, width of midbody pro- and metazonae 0.45 and 0.6 mm (♂), respectively. Length of paratypes 4.0–5.5 mm, width of midbody pro- and metazonae 0.45–0.5 and 0.6–0.7 (♂) or 0.6–0.8 mm (♀), respectively. Coloration in alcohol mostly uniformly reddish, apparently in part due to a thin earth crust coating most of the body (Fig. 1F); more rarely nearly pallid.

Body with 20 segments in both sexes. Tegument very delicately micro-alveolate, slightly shining to dull. Head very densely micropilose, with a very distinct, mushroom-like, frontal tubercle (♂) (Fig. 12D, K). Interantennal isthmus ca 1.3–1.4 times diameter of antennal socket. Antennae long and strongly clavate, reaching back up to segment 3 when stretched dorsally (♂, ♀). In length, antennomere 3 = 6 > 5 > 2 = 4 > 7 > 1; antennomere 6 the largest, antennomeres 5 and 6 each with a distinct, round, distodorsal field of minute sensilla. In width, collum < head < segments 2–4 < 5–16; thereafter body gradually tapering towards telson. Collum ellipsoid, transversely oval, like all following metaterga with three transverse, regular rows of setae. Tergal setae relatively long, each mostly ca 1/3–1.4 as long as metatergum, a little longer on collum and gradually reduced in size towards telson, bacilliform and longitudinally ribbed (Fig. 12A–F, N), always 3+3 in each row on postcollum metaterga. Dorsum invariably regularly convex. Paraterga medium-sized, set at around upper 1/3 of metazonae (Fig. 12A–C), visible starting with collum, often slightly upturned caudally, faintly, but regularly rounded and bordered, lateral incisions absent; but 2–3 setae or their insertion points present at lateral margin. Caudal corner of paraterga mostly rounded, drawn increasingly back, but faintly reaching past rear tergal margin only on segments 18 and 19 (Fig. 12C, F). Pore formula normal: 5, 7, 9, 10, 12, 13, 15–18. Ozopores small, round, opening flush dorsally near caudal corner of poriferous paraterga. Stricture between pro- and metazonae wide, shallow. Limbus very finely microspiculate. Spiracles next to coxae 1 or 2 unusually prominent, discoid and microporose (Fig. 12G, L); following ones small, inconspicuous, as usual. Pleurosternal carinae traceable as very faint ridges or lines on most segments (Fig. 12A–C). Epiproct short, conical, flattened dorsoventrally. Hypoproct semi-circular, setae strongly separated and borne on minute knobs.

Sterna wide, mostly unmodified and sparsely setose, unusually densely setose only between ♂ coxae 2 and 3 (Fig. 12M); each ♂ prefemur 1 with a prominent, densely setose, subtriangular, blunt, distoventral process (Fig. 12J) (much like in *H. falcata*); some setae on ♂ legs slightly modified, with flattened or branching tips. Legs rather long and slender, ca 1.2–1.3 (♂) or 1.0 –1.1 (♀) times as long as midbody height; in length, tarsus > femur > coxa = prefemur = postfemur = tibia, the latter with a particularly long, tactile seta apicodorsally. Tarsal brushes absent.

Gonopods (Fig. 13) with large, subglobose, clearly exposed, alveolate coxae, these rather densely setose nearly throughout, fused medially at base, each carrying two very long setae near place of fusion. Telopodite bases clearly concealed inside a large gonocoel, each very densely setose along funnel-shaped mesal part, with only one strong, slightly curved, very distinctly exposed, ribbon-shaped, apically bird’s beak-shaped branch (ab). Solenomere (sl) a short unciform branch located at and hidden by base of ab.

##### Remarks.

Mfou, the type locality of *Hemisphaeroparia avis* sp. nov., is shared with as many as further two congeners, *H. spiniger* and *H. falcata*.

#### Trichozonus

Taxon classificationAnimaliaPolydesmidaFuhrmannodesmidae

Carl, 1905

C824D654-93F8-54F9-AECF-35D8586429E5

##### Type species.

*Trichozonus escalerae* Carl, 1905, the type species by monotypy, Equatorial Guinea (Carl 1905).

##### Description.

Female. 20 segments, body length 8 mm; paraterga modest, tergal setae long and bacilliform.

##### Remarks.

This genus is bound to remain dubious until a male topotypic sample from Fernando Po becomes available for study. The only other trichopolydesmid known from Fernando Po is *Dendrobrachypus pusillus* Verhoeff, 1941 (= *Mecistoparia pusilla*), which is only 5.0–5.5 mm long (Verhoeff 1941). Mauriès and Heymer (1996) tentatively synonymized *Trichozonus* with *Physetoparia*.

## Discussion

Interestingly, based on the gonopodal conformations alone, all Afrotropical Trichopolydesmidae seem to represent a single lineage characterized by basically rounded, lens-shaped, oblong, relatively small gonotelopodites more or less deeply sunken into a gonocoel and showing, unlike the bulk of Euro-Mediterranean confamilial members (30 species in 17 genera, see Vagalinski et al. (2019)), no transversely oriented bases. The various outgrowths (usually 1–3) of the telopodites, if any, are typically not erect, but curved and directed caudomesad, while the solenomeres, if any, are mostly simple, short, fully mesal processes or lobes. In addition, most species in life tend to show different tinges of red, but are quick to fade in alcohol. Only one genus and species, *Simplogonopus rubellus* (Attems, 1902), also reddish *in vivo*, seems to be of the Afrotropical stock, but it occurs beyond tropical Africa. It has been recorded only from Crete, the Aegean islands of Kythnos and Chios, and northeastern Bulgaria (Vagalinski et al. 2019). Among the possible reasons to explain such a distribution, the following have been considered: (1) a palaeorelict survivor, (2) a human-caused introduction, and (3) recent migration. A combination of reasons cannot be excluded either (Vagalinski et al. 2019).

Previous knowledge of the trichopolydesmid fauna of Cameroon (Golovatch et al. 2018) seems to point to two interesting observations. Cameroon presently appears to be the country in Africa best known with regard to Trichopolydesmidae diversity, even though its trichopolydesmid fauna seems to be surprisingly monotonous, represented by species (16 of 26) of a single large genus, *Hemisphaeroparia*, which ranges from Guinea in the west to Uganda in the east. This has also permitted us to provisionally assign an old species described from Cameroon to that genus as well. The second observation is that there tend to be as many species as localities, meaning that each species has been encountered in a single place. Localities that support two species are rather exceptional (Golovatch et al. 2018).

Our present contribution partly disproves the latter observation, since two already described species have been found more widespread and occurring at least at localities other than the type ones. Moreover, the present paper reveals that one and the same locality can harbour as many as three congeners! It is quite clear that the diversity of Trichopolydesmidae in tropical Africa, despite all efforts, both past and present, remains grossly understudied. Many new taxa and records are undoubtedly still ahead, but we believe we have a sufficiently solid foundation to continue.

## Supplementary Material

XML Treatment for Physetoparia complexa

XML Treatment for Bactrodesmus

XML Treatment for Bactrodesmus grandis

XML Treatment for Hemisphaeroparia parvula

XML Treatment for Hemisphaeroparia falcata

XML Treatment for Hemisphaeroparia spiniger

XML Treatment for Hemisphaeroparia longibrachiata

XML Treatment for Hemisphaeroparia avis

XML Treatment for Trichozonus
